# CircPTK2-miR-181c-5p-HMGB1: a new regulatory pathway for microglia activation and hippocampal neuronal apoptosis induced by sepsis

**DOI:** 10.1186/s10020-021-00305-3

**Published:** 2021-05-05

**Authors:** Min Li, Junwen Hu, Yucong Peng, Jingbo Li, Reng Ren

**Affiliations:** 1 Neuroscience Intensive Care Unit, The Second Affiliated Hospital of Zhejiang University School of Medicine, No. 88, Jiefang Road, Hangzhou, 310009 Zhejiang Province China; 2 Department of Neurosurgery, The Second Affiliated Hospital of Zhejiang University School of Medicine, No. 88, Jiefang Road, Hangzhou, 310009 Zhejiang Province China

**Keywords:** Microglia, Sepsis, CircPTK2, MiR-181c-5p, High mobility group box-1

## Abstract

**Background:**

Circular RNA hsa_circ_0008305 (circPTK2), miR-181c-5p and High mobility group box-1 (HMGB1) had a targeted regulatory relationship through bioinformatics analysis. This study explained the effects of these genes in microglia and sepsis mice.

**Methods:**

Lipopolysaccharide (LPS) or Cecal Ligation and Puncture (CLP) was used to induce inflammation cell model or sepsis mouse model, as needed. Gene levels were measured by enzyme linked immunosorbent assay (ELISA), quantitative real-time PCR or Western blot, as required. Apoptosis was detected by TUNEL assay, and RNase R was used to test the stability of circPTK2. Targeting relationships between genes were analyzed using bioinformatics analysis and dual luciferase assay. Morris water maze test and mitochondrial membrane potential (MMP) detection were conducted to analyze the effects of genes on cognitive dysfunction of mice.

**Results:**

Lipopolysaccharide induction triggered the release of pro-inflammatory cytokines, the upregulation of HMGB1 and circPTK2, and the downregulation of miR-181c-5p in microglia. Overexpression of HMGB1 enhanced the effect of LPS, while silencing HMGB1 partially counteracted the effect of LPS. Moreover, miR-181c-5p was a target of circPTK2 and bound to HMGB1. MiR-181c-5p mimic partially reversed the functions of LPS and HMGB1 overexpression, reduced the levels of TNF-α, IL-1β, and HMGB1, and inhibited apoptosis. CircPTK2 knockdown had the same effect as miR-181c-5p up-regulation. In vivo, sicircPTK2 improved cognitive function, restored MMP level, inhibited apoptosis, reduced the levels of inflammatory factors and apoptotic factors, and increased the survival rate of CLP-induced mice.

**Conclusion:**

Our research reveals that circPTK2 regulates microglia activation and hippocampal neuronal apoptosis induced by sepsis via miR-181c-5p-HMGB1 signaling.

## Background

Sepsis is a serious public health problem worldwide (Rello et al. 2017). In the early stage of sepsis, the nervous system quickly transmits inflammation signals to the central nervous system, and then affects the occurrence and development of sepsis by adjusting the endocrine and immune systems (Rey and Besedovsky 2017). Research shows that the nervous system can also directly regulate the course of sepsis through its own neurotransmitters (Piva et al. 2015). Exploring the pathogenesis of sepsis not only has important theoretical significance, but also may provide new strategies for the treatment of sepsis.

There is convincing evidence that microglia are the main immune effector cells of the central nervous system and play an important role (Kabba et al. 2018). Microglia mediate postoperative hippocampal inflammation and cognitive decline (Feng et al. 2017), and may be responsible for obesity-associated cognitive decline and dendritic spine loss (Cope et al. 2018). High mobility group box-1 (HMGB1) is a non-histone chromosomal binding protein released by necrotic cells (Tsung et al. 2014). It can activate microglia to produce inflammatory factors, and inflammatory cytokines in turn promote the secretion of HMGB1, forming a positive feedback loop which can continuously aggravate the inflammatory response (Wang et al. 2015). This positive feedback effect plays a vital part in maintaining the inflammatory response (Gonelevue et al. 2018). Despite the discovery of the important role of HMGB1 in microglia activation, the mechanism of HMGB1 in sepsis neuroinflammation remains unclear.

MicroRNA (miRNA) is abundantly expressed in the nervous system, and has the characteristics of specificity, timeliness and regionality (Trivedi and Ramakrishna 2009). MiRNAs are not only related to the development of brain tissue, neuronal differentiation and advanced neural functions (such as learning and memory), but also involved in neurodegenerative diseases, mental diseases and other diseases (Ferrante and Conti 2017). In recent years, it has been found that in sporadic Alzheimer’s disease (AD), the down-regulation of miR-137, miR-181c, miR-9, etc. leads to the occurrence of AD (Zhou et al. 2016; Saraiva et al. 2017; Geekiyanage et al. 2012). In addition, aberrant expression of miR-181c partially protects neurons from apoptosis induced by microglia activation through tumor necrosis factor (TNF)-α (Zhang et al. 2012). Given the prediction obtained through bioinformatics in this study that miR-181c-5p bound to HMGB1, we speculated that miR-181c-5p may act against neurological dysfunction caused by sepsis-related encephalopathy through HMGB1.

Circular RNA (circRNA) has a stable loop structure and can be used as a miRNA sponge to regulate gene expressions (Panda 2018). For example, circPTK2 overexpression inhibits TGF-β-induced EMT and invasion of non-small cell lung cancer cells by adsorbing miR-429/miR-200b-3p to target TIF1γ (Peng et al. 2018). Studies have shown that miR-29b binds to circPTK2 and thereby promotes microglial apoptosis (Wang et al. 2019); to our knowledge, however, miR-29b may not be the only downstream target miRNA of circPTK2. In addition, although the above studies have reported the role of circPTK2 in various biological processes, its mechanism of action in sepsis neuroinflammation is also unclear. We found through a miRNA target prediction software that circPTK2 can target miR-181c-5p.

In summary, we explored the role of circPTK2-miR-181c-5p-HMGB1 in hippocampal neuronal apoptosis induced by sepsis via miR-181c-5p-HMGB1 signaling.

## Methods

### Ethics statement

The animal studies were conducted under the approval of the Institutional Animal Care and Use Committee of The Second Affiliated Hospital of Zhejiang University School of Medicine (DS201908027), and following the laboratory guidelines for animal cares.

### Cells and treatment

BV2 microglia were procured from China Center For Type Culture Collection (CCTCC, China), cultured in MEM (PM150410, Procell, China) containing 10% FBS (164210-500, Procell, China) and 1% P/S (PB180120, Procell, China), and placed in a 37 °C, 5% CO_2_ cell incubator (Boxun, BC-J80S, China).

For subsequent studies, lipopolysaccharides (LPS, L4391, Sigma-Aldrich, USA) (0, 25, 50, 100, or 200 ng/ml) was used to induce BV2 microglia for 24 h (h) to establish an inflammatory response model (Tian et al. 2019).

### Cell transfection

The sequence fragment of HMGB1 was amplified and inserted into the pcDNA3.1 (V79520, Invitrogen, USA) vector, named as HMGB1. The pcDNA3.1 vector without target gene sequences was used as NC group. SiRNA interference HMGB1 (siHMGB1, siB11129130839-1-5) was purchased from Guangzhou RIBOBIO Biotechnology Co., LTD (China). SicircPTK2 (A09003, 5′-GUGUCAGAAAAGAU GUUGGUU-3′), siNC (A06001, 5′-CACAGUCAAAAGAUGUUGGUU-3′), miR-181c-5p mimics (B01001, 5′-AACAUUCAACCUGUCGGUGAGU-3′), mimic control (B04001), miR-181c-5p inhibitors (B03001, 5′-ACUCACCGACAGGUUGAAUGUU-3′), and inhibitor control (B04003) were purchased from GenePharma company (China). When grown to reach 70–90% confluence in 24-well plates, BV2 microglia were transfected with different vectors using Lipofectamine 3000 (L3000015, Invitrogen, USA) according to the procedure, as needed.

### Enzyme linked immunosorbent assay (ELISA)

For cell samples, the cells were centrifuged at 200×*g* for 5 minutes (min) and the supernatant was taken for detection. For mouse whole blood samples, the whole blood was allowed to stand at room temperature for 30 min–2 h. After the whole blood naturally coagulated and the serum was precipitated, the sample was centrifuged at about 1000×*g* at 4 °C for 10 min, and the yellow supernatant was taken for use. A Mouse TNF-α ELISA Kit (PT512) and a Mouse interleukin (IL)-1β ELISA Kit (PI301) were ordered from Beyotime Company (China). A Mouse HMGB1 ELISA Kit (MOFI00232) was purchased from Beijing Lebo Biotechnology Co. LTD (China).

### Quantitative real-time PCR

Total RNA isolation from BV2 microglia or mouse tissues was performed using RNAiso Plus (9108Q, Takara, China) or RNAiso for Small RNA (9753Q), as required. Reverse transcription of RNA into cDNA was completed with a PrimeScrip RT reagent Kit (RR037Q, Takara, China). RT-PCR detection was performed in the QuantStudio 5 PCR instrument (ABI, USA) with TB Green Premix Ex Taq II (Tli RNaseH Plus) (RR820Q, Takara, China), under the specific conditions: pre-denaturation (95 °C, 3 min), followed by denaturation (95 °C, 15 s) and annealing (60 °C, 1 min), for a total of 40 cycles, and finally extended (68 °C, 7 min) and stored (4 °C). β-actin or U6 were used as an internal parameter, and the final results were quantified using the 2^−ΔΔCt^ method (Coleman et al. 2017). All the primers were listed as follows (5′–3′). circPTK2: (ATCATACTGGGAGATGCGGG, AGTTGGGGTCAAGGTAAGCA); IL-1β: (GAAACAGCAATGGTCGGGAC, AAGACACGGGTTCCATGGTG); TNF-α: (AGCCCTGGTATGAGCCCATGTA, CCGGACTCCGTGATGTCTAAG); HMGB1: (GCTGACAAGGCTCGTTATGAA, CCTTTGATTTTGGGGCGGTA); Caspase-3: (TGAAGGGGTCATTTATGGGACA, CCAGTCAGACTCCGGCAGTA); β-actin: (GTGACGTTGACATCCGTAAAGA, GCCGGACTCATCGTACTCC); miR-181c-5p: (TCAACCTGTCGGTGAGTGTC, GTATCCAGTGCGTGTCGTGG); U6: (CTCGCTTCGGCAGCACA, AACGCTTCACGAATTTGCGT).

### Target gene prediction and verification

TargetScan v7.2 was used to predict the binding site between HMGB1 and miR-181c-5p. The relationship of miR-181c-5p and circPTK2 was analyzed using starBase v2.0. Dual luciferase assay was used to verify the target relationship between HMGB1 and miR-181c-5p, or miR-181c-5p and circPTK2. The wild-type (HMGB1-WT) or mutant sequences (HMGB1-MUT) of HMGB1 or circPTK2 were inserted into the pmirGLO vector (E1330, Promega, USA) to synthesize reporter plasmids. The reporter plasmids and miR-181c-5p mimic (M) or mimic control (MC) were transfected into BV2 microglia as needed. The dual luciferase system (D0010-100T, Solarbio, China) was used to measure luciferase activity. The luciferase activity of different groups was detected using a GloMax 20/20 detector (Promega, USA).

### TUNEL assay

Cell apoptosis was assessed using the TUNEL kit (C1086, Beyotime, China). In short, a TUNEL test solution was prepared for subsequent use according to the instructions of the kit. BV2 microglia or frozen mouse tissue sections were fixed with 4% paraformaldehyde for 30 min, and then incubated with PBS containing 0.3% Triton X-100 at room temperature for 5 min. Subsequently, 50 μl of the TUNEL detection solution was added to the sample, and incubated at 37 °C in the dark for 60 min. Finally, after being mounted with an anti-fluorescence quenching mounting solution, TUNEL-positive cells were observed under a fluorescent microscope (Zeiss L LSM800, Germany) at × 200 magnification.

### Ribonuclease (RNase) R treatment

For RNase R resistance analysis, total RNA of circPTK2 and PTK2 (2.5 μg) was incubated with RNase-R (10U, R0301, GENESEED, China) at 37 °C for 30 min. Afterwards, the expressions of circPTK2 and PTK2 were measured by RT-qPCR.

### Sepsis induction by cecal ligation and puncture (CLP)

The CLP-induced sepsis mouse model was used as described previously (Denstaedt et al. 2018; Savio et al. 2017). Mice were anesthetized by intraperitoneal injection of 80 mg/kg ketamine and 5 mg/kg xylazine to minimize the pain to the mice. In short, the mice were subjected to a 1–2 cm laparotomy under sterile conditions, followed by cecal mobilization and ligation. Then the cecal was punctured with a 21-gauge needle to induce peritonitis, and finally the incision was closed by suture. All mice were given buprenorphine (0.05 mg/kg) postoperatively twice a day for 2 days.

A total of 75 C57BL/6 mice (SPF, 8–10 weeks, 20–25 g, originally from Guangdong Medical Laboratory Animal Center, placed in a SPF level animal house feeding temperature 21 ± 2 °C; relative humidity 30–70%; light period 12/12 h) were randomly divided into five groups (n = 15 in each group): Sham group, CLP group, CLP + siNC group, CLP + sicirc group, CLP + sicirc + antagomir group. Three days before CLP operation, in the CLP + sicircPTK2 group, CLP + siNC group or CLP + sicirc + antagomir group, circPTK2 siRNA (sicirc, 50 μL), circPTK2 control siRNA (siNC) or circPTK2 siRNA combined with miR-181c-5p antagomir (antagomir, 200 nmol/kg in a final volume of 200 μL) was injected into the lateral ventricle of the mouse brain, respectively. After behavioral experiments, all mice were euthanized by deep anesthesia (Pentobarbital Sodium, 150 mg/kg, intraperitoneal injection) (57-33-0, Sigma, USA). Blood samples and tissue samples were collected for later use.

### Morris water maze test

The Morris water maze (Shanghai Yuyan Scientific Instrument Co., LTD., China) consists of a circular pool (inner diameter: 120 cm, height: 50 cm), a platform (diameter: 6–9 cm, height lifting range: 20–35 cm), and a video acquisition and analysis system. The pool was divided into four quadrants: northeast (NE), northwest (NW), southeast (SE), and southwest (SW) at equal distances on the rim. Appropriate amounts of titanium dioxide were added to the circular pool so that the experimental mice could not see the platform which was located in the center of the SW quadrant. The swimming pool was surrounded by curtains, and the experiment was conducted at a water temperature of 22–26 °C. The Morris water maze test started on the 21th day after treatment in different groups and lasted for 4 days. Four trials were performed each day, and each mouse was put into the pool from the center points of four quadrants for four training. In the first 3 days, the mice received adaptive training. The time to reach the platform was determined as the latency period (i.e. the time from immersing in water to arriving at the platform), with the longest recording time specified as 60 s. The mice were guided to the platform and allowed to stay for 10 s if they failed to enter the platform within 60 s. On day 4, the platform was removed and the video tracking system was used to record the time that the mouse spent in the target quadrant (the original location of the platform) without the platform.

### Mitochondrial membrane potential (MMP) analysis

First, we extracted mitochondria using the tissue mitochondrial isolation kit (C3606, Beyotime, China) to detect the effect of circPTK2 on MMP level. As required, fresh mouse hippocampal tissues that were collected within one hour after the animals were sacrificed were used, and the tissues were kept on ice. Then the measurement of MMP was carried out using the extracted tissue mitochondria and a mitochondrial membrane potential detection kit (JC-1, C2006, Beyotime, China). The final mixed sample was directly subjected to time scan with an F98 fluorescence spectrophotometer (Shanghai Lengguang Technology Co. LTD, China), at an excitation wavelength of 485 nm and an emission wavelength of 590 nm.

### Western blotting (Tian et al. 2019)

The proteins of BV2 microglia or mouse tissues were lysed on ice for 10 min using RIPA lysate (P0013, Beyotime, China), followed by quantification using the BCA method (A53227, ThermoFisher, USA). 20 μg of the protein was subjected to electrophoresis by SDS-PAGE, and then transferred to PVDF membrane (YA1701, Solarbio, China). After being sealed for 1 h, the membrane was incubated with primary antibodies (4 °C, overnight), and then incubated with corresponding secondary antibodies (room temperature, 2 h). Protein bands were visualized with ECL detection reagent (SL1350-100ml, Coolaber, China) and analyzed with gel imaging system (Tanon 2500, Solarbio, China) and Quantity One image analysis software (Bio-Rad, USA). The antibodies used were as follows: IL-1β (ab234437, Abcam, 1/1000, 30 kDa); TNF-α (ab255275, 25 kDa, 1/1000); caspase-3 (1/500, ab32351, 35 kDa); β-actin (ab8226, 1 µg/mL, 42 kDa); HMGB1 (ab79823, 1/10,000, 25 kDa); Goat Anti-Rabbit (ab205718); Goat Anti-Mouse (ab205719).

### Statistical analysis

The data were statistically analyzed using Graphpad prism 8.0 and expressed as mean ± standard deviation. Kaplan–Meier survival analysis was conducted using the log-rank test. The independent sample *t* test was used for comparison between two groups; one-way ANOVA was used for comparison between multiple groups; Bonferroni or Dunnett test was performed for the pairwise comparison as a post hoc test. *P* < 0.05 was considered as statistically significant.

## Results

### LPS induction triggered the release of pro-inflammatory cytokines and the upregulation of HMGB1 in microglia.

**Fig. 1:**
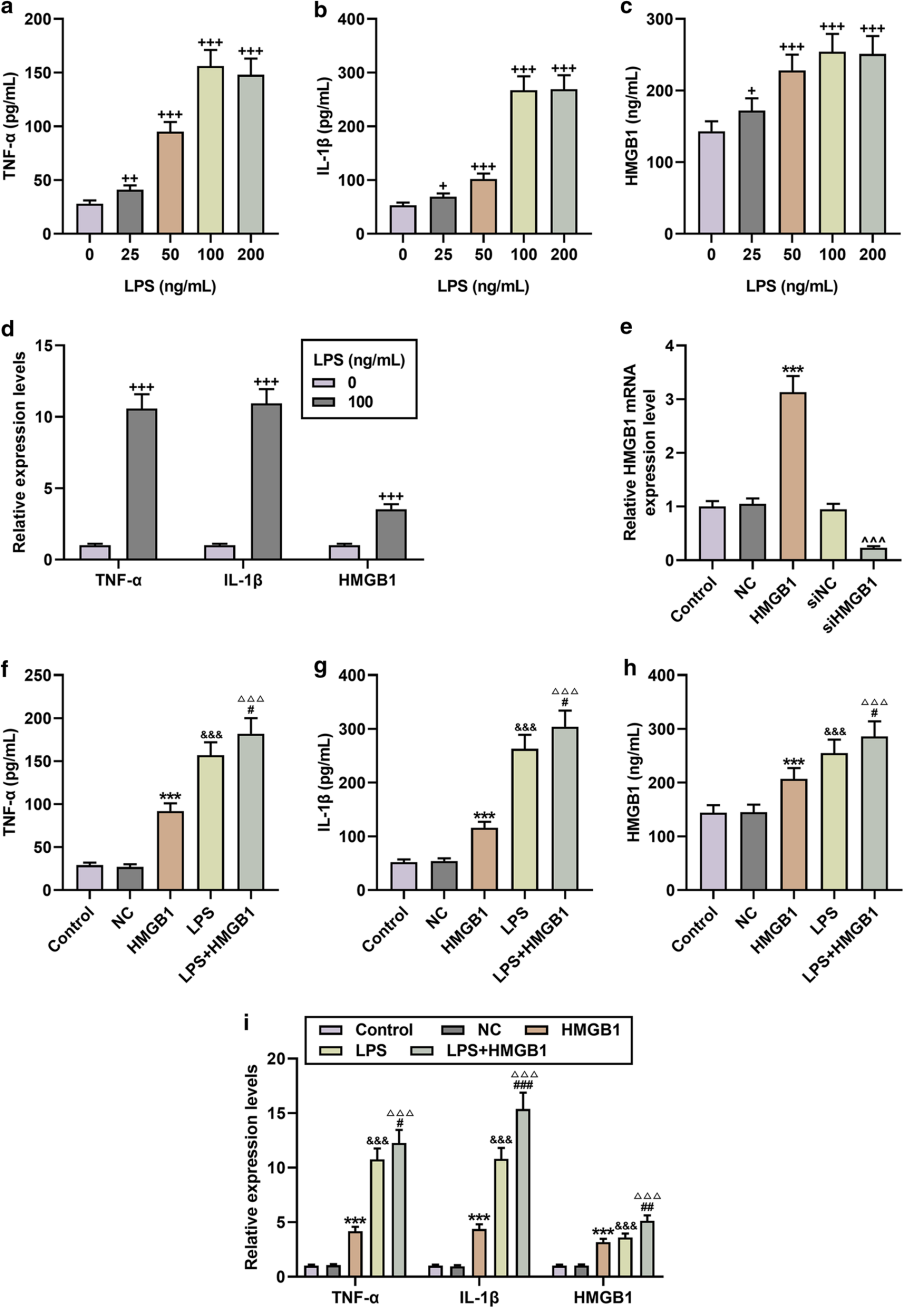
HMGB1 overexpression enhanced the levels of lipopolysaccharides (LPS)-stimulated cytokines in microglia. **a**–**c** After induction with different concentrations of LPS, the contents of TNF-α, IL-1β and HMGB1 in microglia were determined by ELISA. N = 3 for each column; ^+^P < 0.05, ^++^P < 0.01, ^+++^P < 0.001 vs 0; One-way ANOVA followed by Bonferroni's post-hoc test. **d** QRT-PCR was used to detect the expressions of TNF-α, IL-1β, HMGB1 in microglia cells stimulated or not stimulated by LPS. N = 3 for each column; ^+++^P < 0.001 by t test. **e** The expressions of HMGB1 in the Control, Negative Control (NC), HMGB1 (HMGB1 over-expression vector), siNC, and siHMGB1 groups were determined by qRT-PCR. N = 3 for each column; ***P < 0.001, t test vs. NC; ^^^^^P < 0.001, t test vs siNC. (F-I) The results from ELISA and qRT-PCR showed that HMGB1 overexpression promoted the secretion and mRNA expressions of TNF-α, IL-1β and HMGB1, and enhanced the effect of LPS. N = 3 for each column; ***P < 0.001 vs. NC; ^&&&^*P* < 0.001 vs. Control; ^△△△^P < 0.001 vs. HMGB1; ^#^P < 0.05, ^##^P < 0.01, ^###^P < 0.001 vs. LPS; One-way ANOVA followed by Bonferroni's post-hoc test. Each experiment was repeated three times independently. β-actin was used as a control. ELISA: enzyme linked immunosorbent assay; qRT-PCR: quantitative reverse transcription real time polymerase chain reaction

First, we treated BV2 microglia with different concentrations of LPS, and found that after LPS induction, the contents of TNF-α, IL-1β, and HMGB1 in BV2 cells increased significantly, and 100 ng/mL LPS exerted the most obvious regulatory effect (N = 3, *P* < 0.05, Fig. 1a–c). Based on this, we used qRT-PCR to detect the differences in the levels of inflammatory factors and HMGB1 between cells induced by 100 ng/mL LPS and those not induced by LPS. It was found that the levels of TNF-α, IL-1β and HMGB1 increased significantly after LPS induction (N = 3, *P* < 0.001, Fig. 1d).

### HMGB1 regulated the secretion and expressions of TNF-α, IL-1β and HMGB1 in microglia

**Fig. 2:**
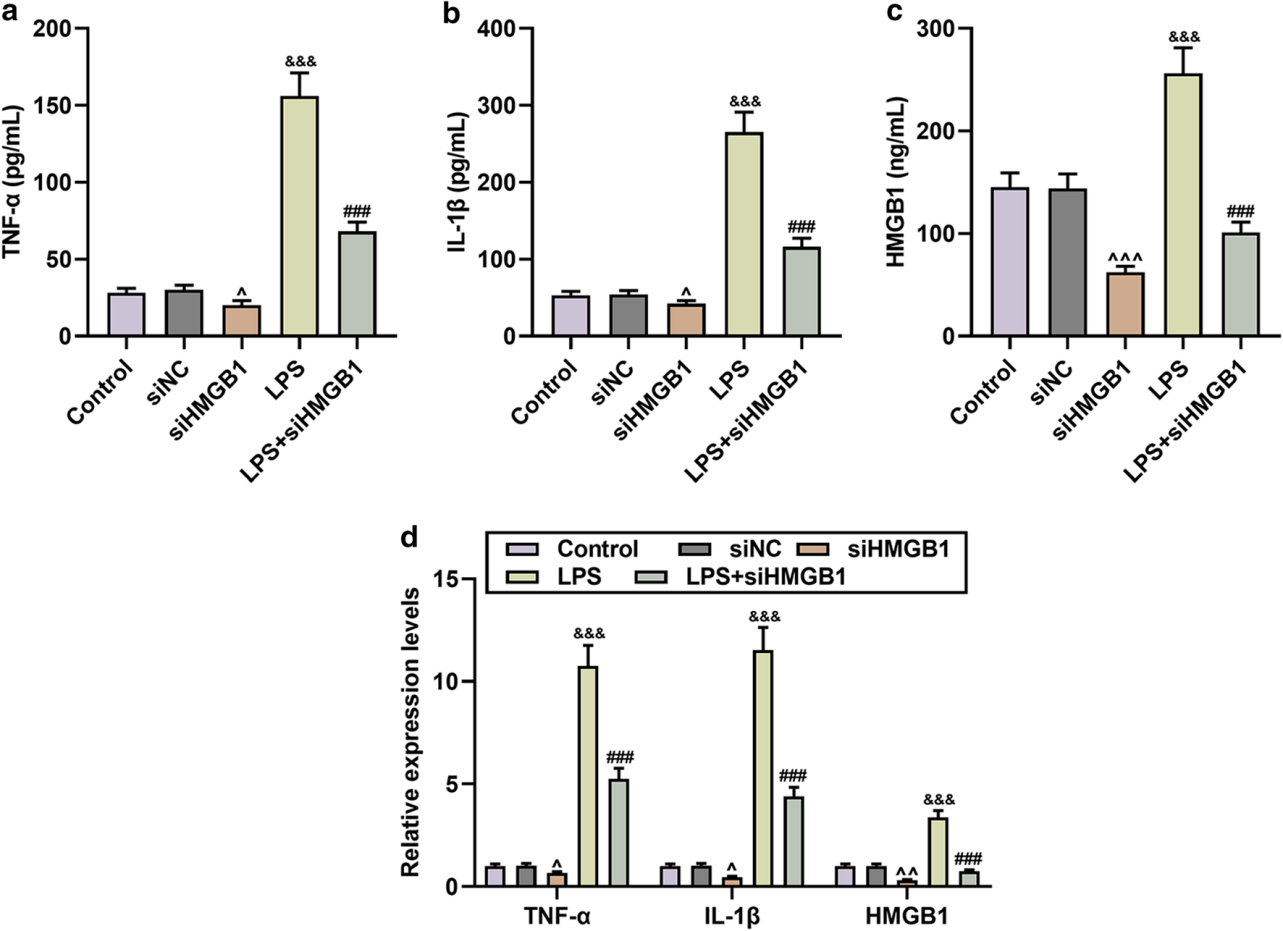
Silencing HMGB1 partially reversed the levels of TNF-α, IL-1β, HMGB1 in LPS-induced microglia. **a**–**c** The contents of TNF-α, IL-1β, and HMGB1 in the Control, siNC, siHMGB1, LPS, LPS + siHMGB1 groups were determined by ELISA. N = 3 for each column; ^^^*P* < 0.05, ^^^^^*P* < 0.001 vs siNC; ^&&&^*P* < 0.001 vs Control; ^###^*P* < 0.001 LPS; One-way ANOVA followed by Bonferroni's post-hoc test. **d** QRT-PCR was used to detect the changes in the mRNA expressions of TNF-α, IL-1β, and HMGB1 in microglia in each group. β-actin was used as a control. N = 3 for each column; ^^^*P* < 0.05, ^^^^*P* < 0.01 vs siNC; ^&&&^*P* < 0.001 vs Control; ^###^*P* < 0.001 vs LPS; One-way ANOVA followed by Bonferroni's post-hoc test. Each experiment was repeated three times independently. *ELISA* enzyme linked immunosorbent assay, *qRT-PCR* quantitative reverse transcription real time polymerase chain reaction

According to reports, HMGB1 plays an important role in neurotoxicity in glial cell activation (Chavan et al. 2012), but its role in sepsis neuroinflammation remains unclear. Therefore, we transfected HMGB1 overexpression vector and siRNA interfered with HMGB1 into microglia. As shown in Fig. 1e, HMGB1 was successfully up-regulated or down-regulated (N = 3, *P* < 0.001). Further research found that overexpression of HMGB1 promoted the secretion and expressions of TNF-α, IL-1β, and HMGB1, and enhanced the effect of LPS (N = 3, *P* < 0.05, Fig. 1f–i). However, HMGB1 knockdown had the opposite effect of HMGB1 overexpression, and partially inhibited LPS-induced microglial activation (N = 3, *P* < 0.01, Fig. 2a–d).

### miR-181c-5p mimic attenuated the cellular inflammation and apoptosis induced by LPS and HMGB1 overexpression

**Fig. 3:**
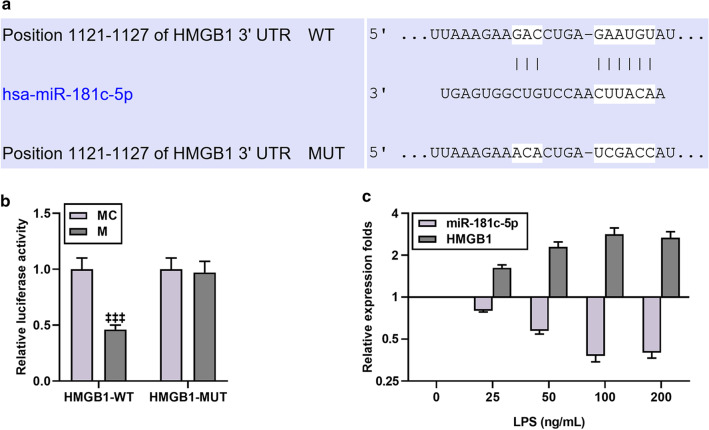
MiR-181c-5p bound to HMGB1 and may have a negative correlation with HMGB1 in LPS-induced microglia. **a** TargetScan v7.2 (http://www.targetscan.org/vert_72/) and **b** dual luciferase assay were used to predict and verify the binding relationship between miR-181c-5p and HMGB1, respectively. HMGB1-WT was the group with wild-type sequence of HMGB1 inserted into a luciferase reporter plasmid. HMGB1-MUT was the group with mutant sequence of HMGB1 inserted into a luciferase reporter plasmid. N = 3 for each column; ^‡‡‡^*P* < 0.001 t test vs MC. **c** After induction with different concentrations of LPS (25, 50, 100, 200 ng/mL), qRT-PCR was used to detect the expressions of miR-181c-5p and HMGB1 in microglia. β-actin or U6 was used as a control. *M* miR-181c-5p mimic, *MC* miR-181c-5p mimic control, *ELISA* enzyme linked immunosorbent assay, *qRT-PCR* quantitative reverse transcription real time polymerase chain reaction

**Fig. 4:**
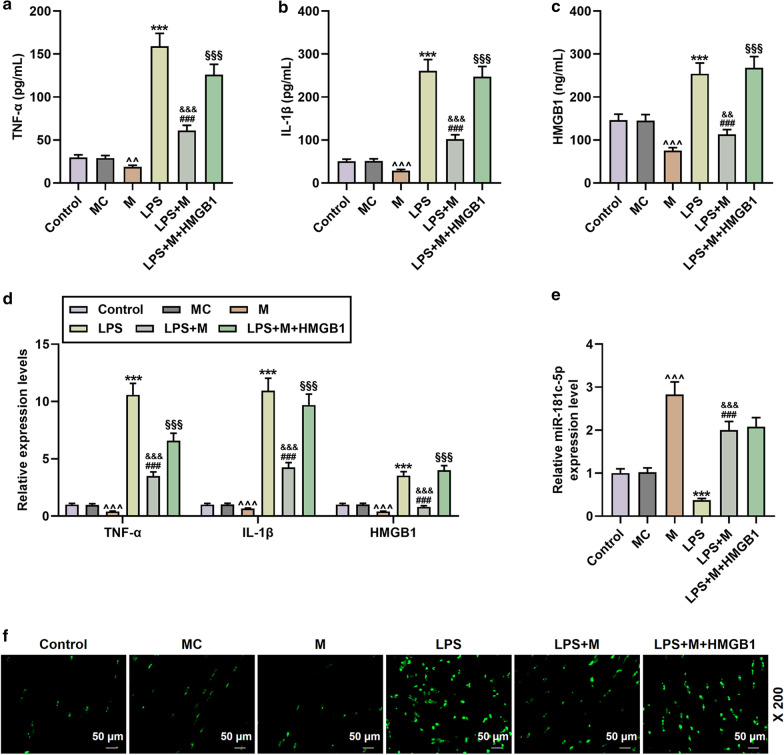
The effect of miR-181c-5p on cytokines and apoptosis in microglia. **a**–**d** The contents and mRNA levels of TNF-α, IL-1β, and HMGB1 in the Control, MC, M, LPS, LPS + M, LPS + M + HMGB1 groups were determined by ELISA and qRT-PCR, respectively. N = 3 for each column; ***P < 0.001 vs Control; ^^^^*P* < 0.01, ^^^^^*P* < 0.001 vs MC; ^&&^*P* < 0.01, ^&&&^*P* < 0.001 vs M; ^###^*P* < 0.001 vs LPS; ^§§§^*P* < 0.001 vs LPS + M; One-way ANOVA followed by Bonferroni's post-hoc test. **e** QRT-PCR was used to determine the level of miR-181c-5p in each group. U6 was served as a control. N = 3 for each column; ***P < 0.001 vs Control; ^^^^*P* < 0.01, ^^^^^*P* < 0.001 vs MC; ^&&^*P* < 0.01, ^&&&^*P* < 0.001 vs M; ^###^*P* < 0.001 vs LPS; ^§§§^*P* < 0.001 vs LPS + M; One-way ANOVA followed by Bonferroni's post-hoc test. **f** The apoptosis of microglia in each group was detected by TUNEL. N = 3. Each experiment was repeated three times independently. *M* miR-181c-5p mimic; *MC* miR-181c-5p mimic control; *ELISA* enzyme linked immunosorbent assay; *qRT-PCR* quantitative reverse transcription real time polymerase chain reaction

As shown in Fig. 3a, miR-181c-5p had a binding site for HMGB1-3′UTR; meantime, the luciferase activity of miR-181c-5p mimic in the HMGB1-WT group was lower than that in the mimic control group, suggesting that miR-181c-5p bound to HMGB1 (N = 3, *P* < 0.001, Fig. 3b). Afterwards, we measured the mRNA expression levels of miR-181c-5p and HMGB1 after treatment with different concentrations of LPS, and as it turned out, the expression level of miR-181c-5p decreased after treatment, while the expression of HMGB1 increased significantly (Fig. 3c). What is more, miR-181c-5p mimic displayed an observable attenuating on LPS-induced inflammation and a down-regulatory effect on HMGB1 content and mRNA levels (N = 3, *P* < 0.01, Fig. 4a–d). Besides, miR-181c-5p mimic also partially restored the miR-181c-5p level inhibited by LPS, whereas overexpression of HMGB1 pose no effect on miR-181c-5p level (N = 3, *P* < 0.001, Fig. 4e). In addition, Fig. 4f showed that compared with the Control group, the amount of apoptosis in the LPS group increased significantly, while that in the M group dropped markedly; moreover, miR-181c-5p mimic reduced LPS-induced apoptosis, whilst this effect was partially reversed by HMGB1 overexpression.

### CircPTK2 regulated the apoptosis of LPS-induced microglia by inhibiting miR-181c-5p

**Fig. 5:**
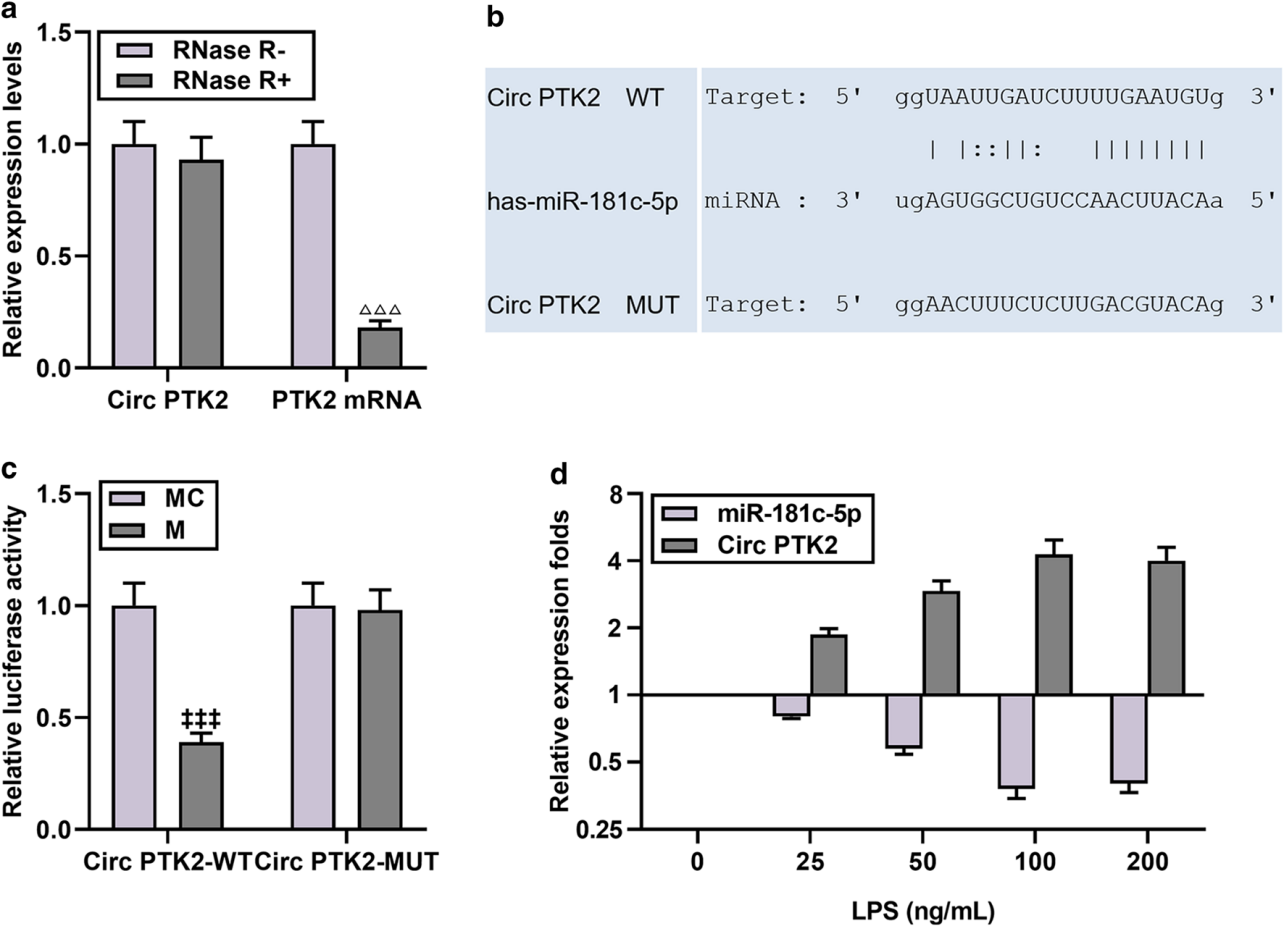
CircPTK2 directly targeted miR-181c-5p and was negatively correlated with miR-181c-5p in LPS-induced microglia. **a** After treatment with RNase R, PTK2 level decreased significantly, while CircPTK2 level remained unchanged. N = 3 for each column; ^△△△^*P* < 0.001 t test vs Rnase R-. **b** The results from starBase v2.0 (http://starbase.sysu.edu.cn/starbase2/) and **c** dual luciferase assay showed that circPTK2 could target miR-181c-5p. N = 3 for each column; ^‡‡‡^*P* < 0.001 t test vs MC. **d** After induction with different doses of LPS, qRT-PCR was used to detect the expressions of miR-181c-5p and circPTK2 in microglia. β-actin or U6 was used as a control. N = 3. Each experiment was repeated three times independently. *M* miR-181c-5p mimic; *MC* miR-181c-5p mimic control; *qRT-PCR* quantitative reverse transcription real time polymerase chain reaction

**Fig. 6:**
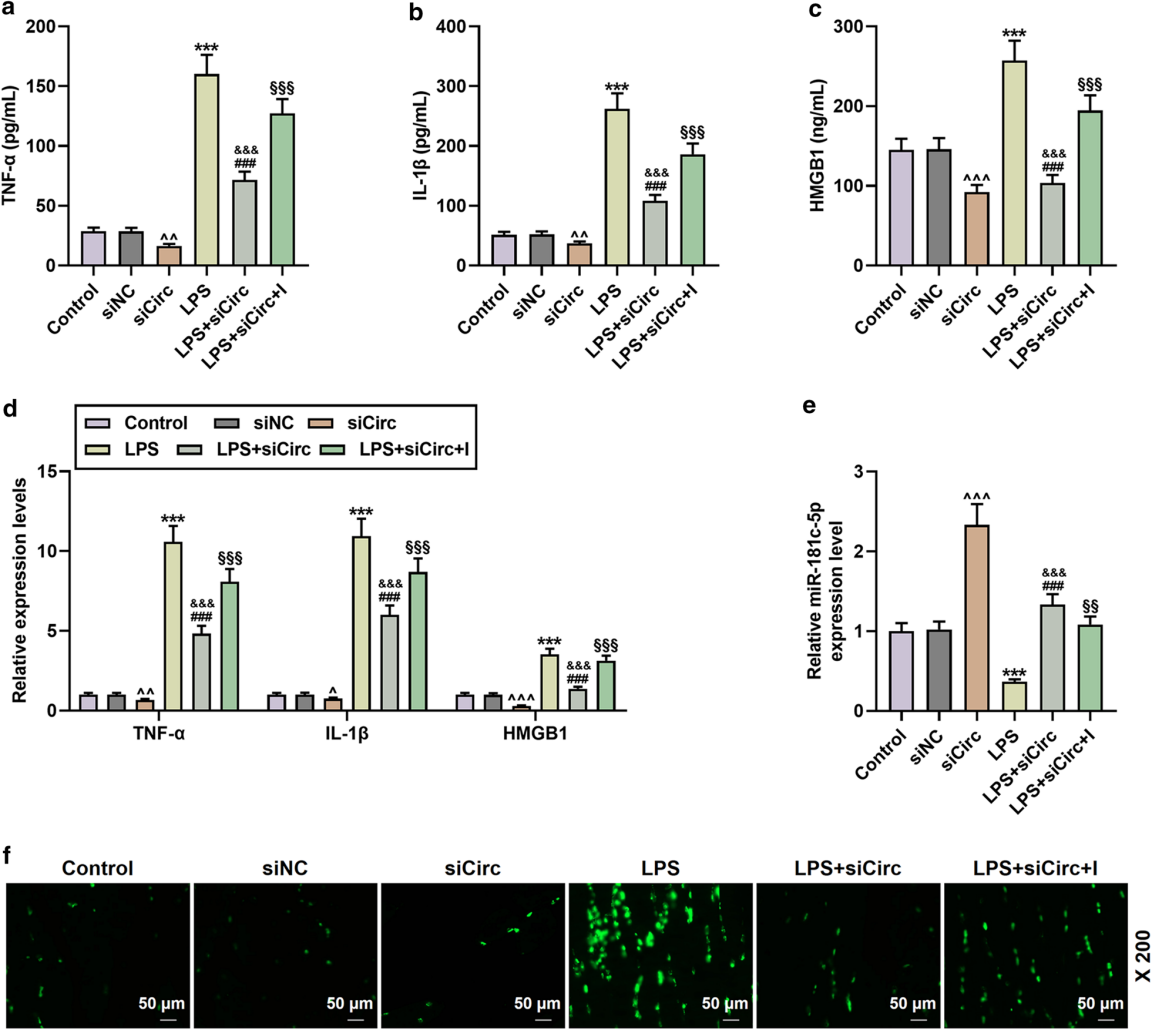
CircPTK2 partially offset the effect of miR-181c-5p on cytokines and apoptosis in microglia. **a**–**d** The contents and mRNA levels of TNF-α, IL-1β, and HMGB1 in the Control, siNC, sicirc, LPS, LPS + sicirc, LPS + sicirc + I groups were detected by ELISA and qRT-PCR. N = 3 for each column; ***P < 0.001 vs. Control; ^^^^*P* < 0.01, ^^^^^*P* < 0.001 vs. siNC; ^&&&^*P* < 0.001 vs. siCirc; ^###^*P* < 0.001 vs. LPS; ^§§§^*P* < 0.001 vs. LPS + siCirc; One-way ANOVA followed by Bonferroni's post-hoc test. **e** QRT-PCR was used to determine the level of miR-181c-5p in each group. N = 3 for each column; ***P < 0.001 vs. Control; ^^^^*P* < 0.01, ^^^^^*P* < 0.001 vs. siNC; ^&&&^*P* < 0.001 vs. siCirc; ^###^*P* < 0.001 vs. LPS; ^§§^*P* < 0.01 vs. LPS + siCirc; One-way ANOVA followed by Bonferroni's post-hoc test. **f** The apoptosis of microglia in each group was detected by TUNEL. *Sicirc* sicircPTK2; *ELISA* enzyme linked immunosorbent assay; *qRT-PCR* quantitative reverse transcription real time polymerase chain reaction. β-actin or U6 were used as control

Studies have shown that circPTK2 promotes glial cell apoptosis, and therefore we further explored its expression in cells (Wang et al. 2019). As shown in Fig. 5a, after RNase R treatment, PTK2 level decreased significantly, while circPTK2 level remained unchanged, proving the stability of circHIPK3 in cells (N = 3, *P* < 0.001). At the same time, through bioinformatics analysis and dual luciferase analysis, we found that circPTK2 targeted miR-181c-5p (Fig. 5b, c). In addition, it was observed that LPS treatment led to an increase in circPTK2 expression (Fig. 5d). Our results also demonstrated that sicircPTK2 lowered the contents and expressions of TNF-α, IL-1β and HMGB1 in LPS-induced microglia, but miR-181c-5p inhibitor partially neutralized the effect of sicircPTK2 (N = 3, *P* < 0.05, Fig. 6a–d). As shown in Fig. 6e, sicircPTK2 increased the miR-181c-5p level in the LPS group, yet LPS + siCirc + I partially suppressed miR-181c-5p expression (N = 3, *P* < 0.01). In addition, sicircPTK2 inhibited LPS-induced apoptosis, but LPS + sicircPTK2 + I increased the amount of apoptosis (Fig. 6f).

### Effect of circPTK2-miR-181c-5p signal on learning and memory functions and apoptosis in CLP-induced mice

**Fig. 7:**
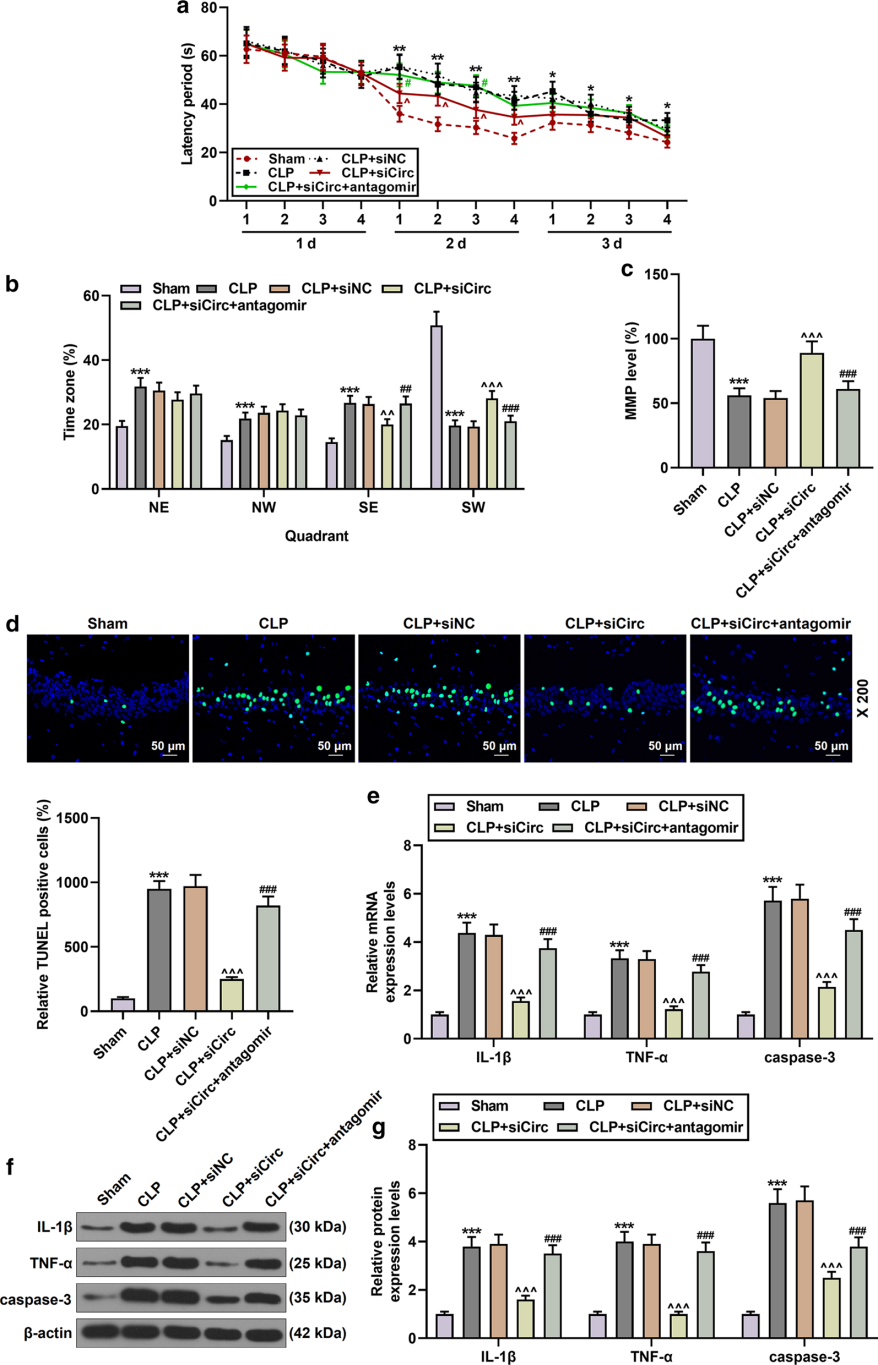
Effects of circPTK2 and miR-181c-5p on learning and memory functions, mitochondrial function, hippocampal cell apoptosis and inflammation in CLP-induced mice. **a** and **b** Morris water maze test was used to measure the latency period and all quadrant residence time of mice in the Sham, CLP, CLP + siNC, CLP + sicirc, CLP + sicirc + antagomir groups. N = 6 per group; *P < 0.05, **P < 0.01, ***P < 0.001 vs. Sham; ^^^*P* < 0.05, ^^^^*P* < 0.01 vs. CLP + siNC^; #^*P* < 0.05, ^##^*P* < 0.01 vs. CLP + siCirc; One-way ANOVA followed by Bonferroni's post-hoc test. **c** CircPTK2 knockdown partially restored mitochondrial membrane potential in CLP-induced mice, and miR-181c-5p antagomir again reversed the effect of sicircPTK2. N = 3 for each column; ***P < 0.001 vs. Sham; ^^^^^*P* < 0.001 vs. CLP + siNC; ^###^*P* < 0.001 vs. CLP + siCirc; One-way ANOVA followed by Bonferroni's post-hoc test. **d** The effects of circPTK2 and miR-181c-5p on hippocampal cell apoptosis was determined by TUNEL. N = 3 for each column; ***P < 0.001 vs. Sham; ^^^^^*P* < 0.001 vs. CLP + siNC; ^###^*P* < 0.001 vs. CLP + siCirc; One-way ANOVA followed by Bonferroni's post-hoc test. **e**–**g** The expressions of IL-1β, TNF-α and Caspase-3 in the hippocampus of mice in each group were detected by qRT-PCR and Western blot. N = 3 for each column; ***P < 0.001 vs. Sham; ^^^^^*P* < 0.001 vs. CLP + siNC; ^###^*P* < 0.001 vs. CLP + siCirc; One-way ANOVA followed by Bonferroni's post-hoc test. *CLP* Cecal Ligation and Puncture; *Sicirc* sicircPTK2; *qRT-PCR* quantitative reverse transcription real time polymerase chain reaction. β-actin were used as control

After investigating the effect of circPTK2-miR-181c-5p-HMGB1 in microglia through the previous experiments, we further constructed a CLP-induced sepsis mouse model. It was found that the latency period of mice in the CLP group increased compared with the Sham group; the latency period of mice in the CLP + sicirc group decreased compared with the CLP group (N = 6, *P* < 0.05, Fig. 7a). Then a probe test was done in the absence of the platform and after the animals had learnt the correct place for the platform in the pool. Conversely, the residence time in NE, NW and SE quadrants of mice was prolonged in the CLP group, while the residence time in SW quadrants of mice was shortened in the CLP group and prolonged in the CLP + sicirc group (N = 6, *P* < 0.001, Fig. 7b). In addition, miR-181c-5p antagomir partially reversed the therapeutic effect of sicircPTK2 (N = 6, *P* < 0.05, Fig. 7a, b). The apoptosis of a variety of cells induced by diverse factors are accompanied by a decline in MMP. Therefore, we also detected the effects of circPTK2 and miR-181c-5p on MMP. It was found that MMP level was lowered in the CLP group; meantime, sicircPTK2 partially restored MMP level, but miR-181c-5p antagomir again caused the opposite effect (N = 6, *P* < 0.001, Fig. 7c). Through further examination of the apoptosis of hippocampus cells in each group, we observed that compared with the Sham group, the number of apoptosis-positive cells in the CLP group increased significantly; however, the CLP + siCirc group showed a decreased number of apoptosis-positive cells, while miR-181c-5p antagomir performed the opposite function of CLP + siCirc (Fig. 7d). In mouse hippocampus, the mRNA and protein levels of IL-1β, TNF-α, and caspase-3 in the CLP group were significantly increased, while sicircPTK2 partially counteracted the up-regulatory effect of CLP on these genes; interestingly, miR-181c-5p antagomir partially reversed the regulatory effect of sicircPTK2 on the above genes (N = 6, *P* < 0.001, Fig. 7e–g). The number of mice detected was 6 in each group.

### Regulation of circPTK2-miR-181c-5p-HMGB1 in CLP-induced mice

**Fig. 8:**
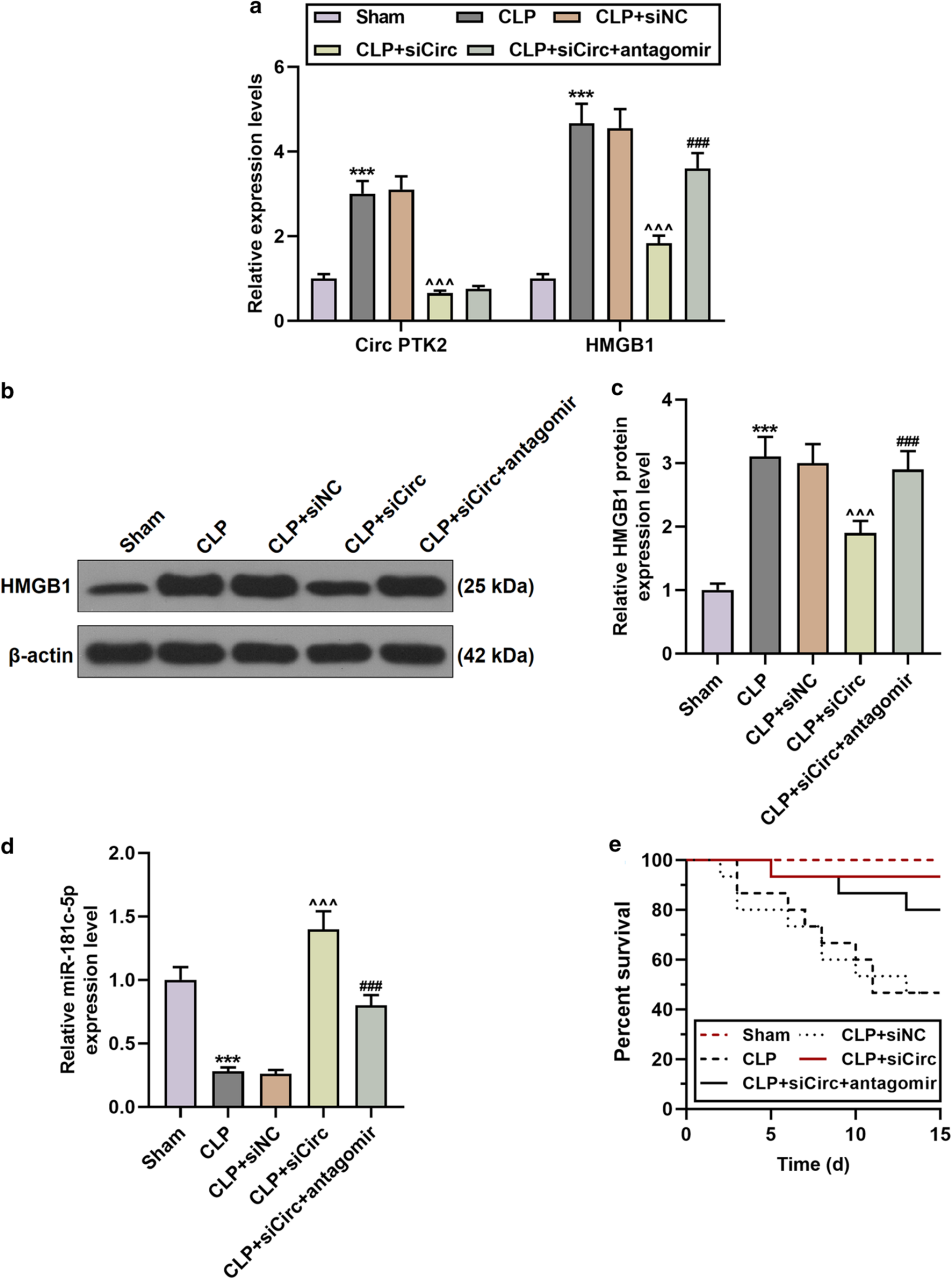
The relationship between circPTK2, miR-181c-5p and HMGB1 in CLP-induced mice. **a**–**d** The expressions of circPTK2, miR-181c-5p and HMGB1 in the hippocampus tissues of mice in the Sham, CLP, CLP + siNC, CLP + sicirc, CLP + sicirc + antagomir groups were detected by qRT-PCR and Western blot. N = 3 for each column; ***P < 0.001 vs. Sham; ^^^^^*P* < 0.001 vs. CLP + siNC; ^###^*P* < 0.001 vs. CLP + siCirc; One-way ANOVA followed by Bonferroni's post-hoc test. **e** Kaplan–Meier curve was used to calculate the survival rate of mice in each group. N = 15. *CLP* Cecal Ligation and Puncture; *Sicirc* sicircPTK2; *qRT-PCR* quantitative reverse transcription real time polymerase chain reaction. β-actin or U6 were used as control

From Fig. 8a–d, we also found that in mouse hippocampus, circPTK2 and HMGB1 were up-regulated and miR-181c-5p was down-regulated in the CLP group compared with the Sham group (N = 6, *P* < 0.001). Compared with the CLP group, the CLP + siCirc group exhibited a decrease in the expressions of circPTK2 and HMGB1 and an increase in miR-181c-5p expression (N = 6, *P* < 0.001). In contrast to the CLP + siCirc group, HMGB1 was up-regulated, miR-181c-5p was down-regulated (N = 6, *P* < 0.001), and circPTK2 expression remained unchanged in the CLP + siCirc + antagomir group. In addition, as shown in Fig. 8e, the CLP group exhibited a reduced survival rate of mice, and sicircPTK2 increased the survival rate of CLP-induced mice; however, the survival number of mice in the CLP + siCirc + antagomir group after 21 days was significantly smaller than that the CLP + siCirc group.

## Discussion

The pathophysiological process of sepsis is very complicated, including inflammation, immunity and neurological dysfunction and other aspects, and involving various changes in cell function, metabolism and microcirculation (Rello et al. 2017; Piva et al. 2015). At present, however, there is still few study on the related regulatory mechanisms of sepsis occurrence and development.

It is commonly known that microglia are suitable for studying neurological dysfunction caused by sepsis-related encephalopathy (Deng et al. 2013). Therefore, we also chose microglia as the object of the study. In addition, LPS, a powerful inflammatory response inducer, is widely used as an inflammatory source to stimulate inflammation in the brain (Noh et al. 2014). Base on this finding, in this study, we used LPS to build a model of microglia inflammation. Microglias are activated during injury and other pathological conditions including LPS (Orihuela et al. 2016). The activated microglia can play a neuroprotective role by phagocytosing necrotic cell fragments and other harmful substances to reduce the subsequent damage caused by necrotic substances on neurons; what is more, they can also produce a series of inflammatory response factors (TNF-α, IL-1β, etc*.*), thereby causing inflammatory cascade in the central nervous system to exert neurotoxicity (Suzumura 2013; Block et al. 2007). In this study, it was found that after LPS stimulation, BV2 cells were activated, which subsequently promoted the up-regulation of TNF-α and IL-1β levels and HMGB1 expression. At the same time, we confirmed the positive feedback loop of HMGB1 and inflammation, which is consistent with previous reports. HMGB1 siRNA partially reversed the secretion of TNF and IL1-beta after LPS challenge in BV2 microglia, which might depended on the incomplete silencing.

Although HMGB1 has been found to cause neuroinflammation by activating microglia, the role of HMGBA as an upstream regulatory pathway has not been confirmed. Therefore, after determining the relationship between HMGB1 and miR-181c-5p, we studied the relationship of the changes in miR-181c-5p expression with proinflammatory cytokines and apoptosis. It was confirmed that up-regulation of miR-181c-5p can lead to a decrease in the release of microglia inflammatory factors, inhibit the apoptosis of TUNEL cells, and down-regulate the expression of HMGB1. In addition, we found that overexpression of HMGB1 did not increase the level of miR-181c-5p, confirming that miR-181c-5p is an upstream regulatory gene of HMGB1. According to previous research, miR-181c-5p has certain functions in cancer, endothelial dysfunction, inflammation and other aspects, yet the functions are not necessarily consistent (Gao et al. 2018; Manzano-Crespo et al. 2019). For example, Ge et al*.* found that up-regulation of miR-181c-5p can aggravate hypoxia/reoxygenation-induced cell damage and promote apoptosis (Ge et al. 2019). MiR-181c-5p can increase the production of malondialdehyde and reactive oxygen species, promote the apoptosis of HUVECs, and significantly enhance high glucose-induced oxidative stress damage (Shen et al. 2018). Overexpressed miR-181c-5p inhibits the inflammatory response in rats with irritable bowel syndrome by silencing IL1A (Ji et al. 2019). The inconsistent function of miR-181c-5p on apoptosis might depend on different cells and mechanisms, which needs further research.

Mounting evidence indicates that circRNA is of great significance to various biological processes (Bao et al. 2019). In sepsis, Nie et al*.* found 373 up-regulated and 428 down-regulated circRNAs using LPS-induced rat sepsis model (Nie et al. 2020). In this study, we found that circPTK2 can target the negative regulation of miR-181c-5p, and thus, we boldly speculated that the three genes circPTK2, miR-181c-5p and HMGB1 may form an inflammation regulation feedback loop in microglia. As expected, circPTK2 silencing attenuated the inflammatory response of cells and suppressed the proliferation of TUNEL-positive cells. These results provided a preliminary theoretical support for our conjecture. The function of circPTK2 has also been reported by other scholars, while it varies in different studies (Yang et al. 2020); for instance, overexpression of circPTK2 promotes the proliferation of gastric cancer cells and tumor growth by adsorbing miR-139-3p (Yu and Zhang 2020).

Corresponding to in vitro experiments, we used a mouse model of sepsis induced by CLP to further study the effects of circPTK2, miR-181c-5p and HMGB1 on mouse hippocampal neuronal apoptosis and cognitive dysfunction. We found that silencing circPTK2 can restore the learning and memory functions of mice with CLP-induced sepsis and increase the survival rate of CLP mice. In addition, when sepsis occurs, mitochondrial dysfunction can result in an insufficiency in the energy that the cells require, which will gradually cause multiple organ failure (Singer 2014). The current study clarified that sicircPTK2 can restore the MMP level decreased by CLP. Similarly, consistent with the in vitro results, our findings revealed that sicircPTK2 can reduce hippocampal cell apoptosis and inhibit the expressions of inflammatory factors and apoptotic factors. As circPTK2 deficient mice are not available, replicating the findings in circPTK2 deficient mice could not be conducted now, which would be further studied in future.

## Conclusions

To sum up, our research demonstrated circPTK2-miR-181c-5p-HMGB1 as a new regulatory pathway in LPS-induced microglia inflammation model and CLP-induced sepsis mouse model. Since HMGB1 deletion is lethal, circPTK2 could be the target in therapeutics for sepsis-induced cognitive dysfunction.

## Data Availability

The analyzed data sets generated during the study are available from the corresponding author on reasonable request.

## Abbreviations

LPS: Lipopolysaccharides
CLP: Cecal ligation and puncture
ELISA: Enzyme linked immunosorbent assay
MMP: Mitochondrial membrane potential
HMGB1: High mobility group box-1
AD: Alzheimer’s disease
TNF: Tumor necrosis factor
circRNA: Circular RNA
